# Racial-ethnic diversity in ambulatory blood pressure monitoring in children with chronic kidney disease

**DOI:** 10.1007/s00467-022-05659-2

**Published:** 2022-07-08

**Authors:** Reut Pagi, Ora Yadin, Katherine Wesseling-Perry, Keith Norris, Marciana Lee Laster

**Affiliations:** 1 Department of Pediatrics, Division of Nephrology, David Geffen School of Medicine at University of California, Los Angeles, 10833 Le Conte Avenue, MDCC A2-383, Los Angeles, CA 90095-1752 USA; 2 Department of Medicine, Division of Nephrology, David Geffen School of Medicine at University of California, Los Angeles, Los Angeles, CA USA

**Keywords:** Chronic kidney disease, Ambulatory blood pressure monitoring, Ethnic groups

## Abstract

**Background:**

Black adults with chronic kidney disease (CKD) have higher rates of hypertension as compared to White adults with CKD. Little is known of how race and ethnicity associate with the prevalence of hypertension in pediatric CKD patients. The aim was to compare ambulatory blood pressure monitoring (ABPM) results for patients with CKD enrolled in the Chronic Kidney Disease in Children (CKiD) study across racial-ethnic groups.

**Methods:**

Patients from the CKiD study who identified as non-Hispanic White, non-Hispanic Black, or Hispanic were included to analyze differences in ABPM results across these racial-ethnic groups. The outcomes were fitted using 3 progressively adjusted models.

**Results:**

This study included 501 CKiD participants with at least one successful ABPM study. Compared to White participants, Black participants had 4.2 mmHg higher mean sleep systolic blood pressure and 2.7 mmHg higher mean sleep diastolic blood pressure (*p* = 0.001 and *p* = 0.004, respectively). Additionally, Black participants had higher odds of abnormal wake systolic load (*OR* 1.88, 1.21–2.91, *p* = 0.005), wake diastolic load (*OR* 1.68, 1.03–2.73, *p* = 0.04), sleep systolic load (*OR* 2.19, 1.36–3.5, *p* = 0.001), sleep diastolic load (*OR* 2.01, 1.28–3.15, *p* = 0.002), systolic non-dipping (*OR* 2.02, 1.31–3.10, *p* = 0.001), and diastolic non-dipping (*OR* 2.69, 1.60–4.51, *p* < 0.001). Compared to White participants, Hispanic participants demonstrated only a lower sleep diastolic load (*OR* 0.54, 0.31–0.95, *p* = 0.03).

**Conclusions:**

Black children with CKD have higher absolute nocturnal blood pressures and higher rates of abnormal dipping. Further studies are needed to determine the etiology of these differences and the clinical implications of racial-ethnic differences in ABPM outcomes within the pediatric CKD population.

**Graphical abstract:**

A higher resolution version of the Graphical abstract is available as Supplementary information

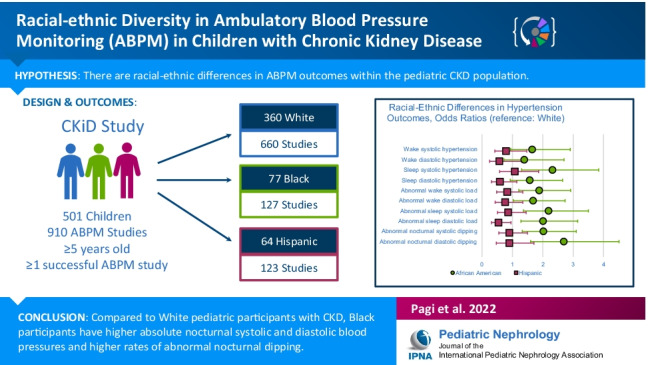

**Supplementary Information:**

The online version contains supplementary material available at 10.1007/s00467-022-05659-2.

## Introduction

Hypertension remains a major public health challenge and is an extensively studied risk factor for cardiovascular disease in both pediatric and adult populations. In the adult population, the rate of mortality from heart disease or stroke doubles for every 20 mmHg increase in systolic blood pressure and 10 mmHg increase in diastolic blood pressure above 140/90 mmHg [1]. Within the pediatric population, the global prevalence of hypertension is as high as 4% and is a major contributor to long-term morbidity and mortality [2]. Pediatric hypertension is predictive of adult hypertension and is correlated with risk factors for cardiovascular events including left ventricular hypertrophy, early atherosclerosis, and microalbuminuria [3]. Over time, the importance of blood pressure phenotypes *not* reflected by in-office blood pressure measurements has become evident. These diagnoses include masked hypertension, nocturnal hypertension, and white-coat hypertension, all of which require the use of ambulatory blood pressure monitoring (ABPM), the preferred method for the diagnosis and monitoring of hypertension.

Hypertension is highly prevalent in adult and pediatric patients with chronic kidney disease (CKD). The prevalence of hypertension in adults with CKD ranges between 60 and 90% and in the pediatric population between 50 and 80%, depending on the CKD stage [4]. As such, Samuels et al. explored the trends of hypertension diagnosed by ABPM and disease progression in the pediatric patients enrolled in the Chronic Kidney Disease in Children (CKiD) study and found that 58% of patients had abnormal ABPM results [5]. Furthermore, a study by Mitsnefes et al*.* showed that 38% of CKiD patients had masked hypertension, and that abnormal ABPM was associated with left ventricular hypertrophy [6].

It has been shown that the prevalence of clinic-defined hypertension and ABPM-defined hypertension differs according to racial and ethnic group. For instance, the Coronary Artery Risk Development in Young Adults (CARDIA) study demonstrated a higher prevalence of abnormal ambulatory blood pressure monitoring in Black participants as compared to White participants [7]. More specifically, the CARDIA study demonstrated racial differences in nocturnal blood pressure parameters including increased rates of nocturnal systolic and diastolic hypertension as well as blunted systolic and diastolic nocturnal dipping in Black participants as compared to White participants [8]. In a longitudinal study from childhood to early adulthood, Wang et al. demonstrated a similar predilection for racial differences in nocturnal hypertension and estimated that these differences are present as early as 10 years of age [9].

Additionally, the increasing prevalence of pediatric hypertension seen in recent decades is more pronounced in Black and Hispanic children and adolescents [10–12]. At present, little is known of how race and ethnicity associate with the prevalence of hypertension in pediatric CKD patients. Therefore, we aim to compare ambulatory blood pressure monitoring results for patients with CKD disease enrolled in the Chronic Kidney Disease in Children (CKiD) study across racial-ethnic groups.

## Methods

### Study population

Data from this study were obtained from the NIH/NIDDK data repository of the CKiD study [13]. The CKiD study is an NIH-funded, prospective, observational, multicenter study. The CKiD study was initiated in 2005 and collects data from chronic kidney disease patients starting at age 6 months to 16 years at over 50 pediatric nephrology centers in North America. The study was designed to evaluate risk factors for progression and the effects of progression of chronic kidney disease. Details of the CKiD study design have been previously published [14]. The CKiD study protocol was reviewed and approved by each participating center’s institutional review board.

For our study, we included patients older than 5 years of age, who had one or more successful ABPM studies and who identified as non-Hispanic White (hereafter referred to as White), non-Hispanic Black (hereafter referred to as Black), and Hispanic. Patients less than 5 years of age were excluded given the decreased reliability of ABPM in this age group. The time period of study entry for these patients was between 2005 and 2014. The latest year of follow-up in this cohort was 2016.

### Study variables

The primary exposure of interest was self-reported racial-ethnic group. Participants in the CKiD study or patient representatives reported the racial-ethic group with which the patient identified and are categorized as White, Black, and Hispanic for the purposes of this study. Given the small patient population, participants of other races including Asian, Native-American, and mixed race were excluded from the analysis. CKD etiology was classified as glomerular and non-glomerular. The glomerular category included primary and secondary focal segmental glomerulosclerosis (FSGS) and the non-glomerular category included congenital anomalies of the kidney and urinary tract (CAKUT), cystic disorders, and hereditary disorders. Estimated glomerular filtration rate (eGFR) was calculated according to the bedside Schwartz equation and used to define CKD stages [15].

Ambulatory blood pressure monitoring (ABPM) occurred for study participants 1 year after study entry and was repeated every 2 years thereafter. SpaceLabs® 90,217 monitors (SpaceLabs® Healthcare, Issaquah, WA) were used for all ABPM. Monitors were sent from the ABPM center at the University of Texas Health Science Center in Houston to the CKiD study site and then returned to the ABPM center for analysis. All of the clinical sites received training in monitor placement and measured participant’s arm circumference for appropriate cuff size selection. Ambulatory blood pressure monitors were worn for 24 h and measured blood pressure (BP) every 20 min during both day and night. Each participant self-reported periods of sleep, wake, and times of medication administration.

A successful ABPM study was defined as one where the monitor was worn ≥ 21 h, with ≥ 18 h with at least 1 recording per hour. Each study had to have at least 1 successful blood pressure measurement in ≥ 75% of wake and sleep hours. Ambulatory blood pressure monitoring parameters of interest were mean wake systolic and diastolic measurements and mean sleep systolic and diastolic measurements. Wake and sleep systolic and diastolic hypertension were defined as blood pressure ≥ 95th percentile for height and gender using Soergel et al.’s ambulatory blood pressure values in healthy children [16]. Ninety-fifth percentiles as defined by Wuhl et al. were unavailable in the publicly available cohort [17]. Abnormal load was defined as > 25% abnormal readings during awake and sleep periods. Nocturnal dipping was calculated as the percent decrease from mean wake to mean sleep systolic and diastolic blood pressure, and abnormal dipping was defined as < 10% decline in blood pressure between the awake and asleep state.

### Statistical analysis

Baseline characteristics were reported as means and standard deviations, and differences in continuous baseline characteristics between racial ethnic groups were compared using analysis of variance (ANOVA). The chi-square test was used to compare categorical variables, presented as total, *n*, and percentages, and the Fischer exact test where appropriate for categories less than 5.

Linear mixed effects models were used for the continuous outcomes, wake and sleep mean systolic, and diastolic blood pressure, and generalized estimating equation models for the remaining categorical outcomes. Models were sequentially adjusted for relevant covariates, and the White cohort was used as the reference group. Model 1 included race-ethnicity, age, and gender. Model 2 was additionally adjusted for body mass index (BMI), height z score, CKD stage, and CKD etiology (glomerular vs. non-glomerular) and Model 3 was additionally adjusted for antihypertensive use (yes or no).

The impact of time from baseline CKiD visit was considered for each model and was only significant for the wake systolic hypertension categorical outcome. Since time was significant for this outcome, it was included in all of its 3 models. Due to missing data on height and weight in addition to age at time of ABPM over 20 years, BMI z score could not be imputed on 46 participants. Therefore, absolute BMI was used in the primary models and a sensitivity analysis was performed to determine the impact of using BMI z score or obesity, defined as BMI ≥ 95th percentile, in the population with BMI z score availability. Additional sensitivity analyses were conducted to determine the impact of using eGFR vs. CKD stage and to determine whether or not results differed when analyzing the first available test in simple linear regression as opposed to all available tests in mixed effects models. A *p* value of < 0.05 was considered significant. Data was analyzed using SAS Studio statistical software.

## Results

### Baseline patient characteristics

At the time of collection from the public database, 891 patients were enrolled in the CKiD study, 696 of which had ABPM studies. Of these, only 622 patients had successful studies. We narrowed our cohort to patients of Black, White, and Hispanic ethnic groups. We then excluded 7 patients < 5 years of age with the assumption, based on clinical experience, that ABPM studies in very young children may be inaccurate. Our study population consisted of 501 remaining patients—360 White, 77 Black, and 64 Hispanic patients (Supplemental Figure 1). Of the final population, 45% of the study patients had only 1 ABPM study, 36% had 2 ABPM studies, 12% had 3 ABPM studies, 6% had 4 ABPM studies, and only 1% had 5 ABPM studies. ABPM use was less common in Black participants in whom 69% of all participants had at least one ABPM study in contrast to 82% of White participants and 79% of Hispanic participants (*p* = 0.004). Additionally, of those with an ABPM study, only 48% of Black patients had more than one ABPM study as compared to 55% of White participants and 64% of Hispanic participants.

The mean age of the patient cohort at the baseline visit was 12.7 years with 39% female and 61% male patients. There was no significant difference among the racial-ethnic groups in age or gender (Table 1). Twenty-five percent of the patients had a glomerular cause of CKD and 75% had non-glomerular causes of CKD. The White cohort had fewer patients with glomerular CKD (21%) as compared to the Black cohort (38%) and the Hispanic cohort (30%). The mean eGFR was 51.6 ml/min/1.73 m^2^; however, the Hispanic cohort had the lowest mean eGFR (44.2 ml/min/1.73 m^2^). There was no difference in frequency of CKD stages among the groups, with almost half of the patients having CKD stage 3. The mean body mass index (BMI) was highest in the Black participants (22.5 kg/m^2^), lowest in White participants (20.0 kg/m^2^), and intermediate in Hispanic participants (21.1 kg/m^2^) (Table 1). The prevalence of obesity differed among the groups (overall *p* value = 0.006) and was higher in Black participants when compared to White participants (pairwise *p* value = 0.001) but not different between Hispanic and White participants (pairwise *p* value = 0.3). There was no difference in height z-scores. The percent of patients prescribed anti-hypertensives did not differ among the racial-ethnic groups, with 66% of patients in the cohort prescribed anti-hypertensives. Furthermore, there was no significant difference in the use of angiotensin converting enzyme inhibitors (ACEI) or angiotensin receptor blockers (ARB) yet the frequency of ACEI or ARB use was lowest in Black children.

**Table 1:** Data at first ABPM by racial-ethnic group

	Total	Black	White	Hispanic	*P* value*
*N* (% total)	501 (100)	77 (15.4)	360 (71.9)	64 (12.8)	
Age at time of study in years, mean (SD)	12.7 (3.8)	12.6 (4)	12.66 (3.8)	12.8 (3.9)	1
Gender					0.07
Female, *n* (%)	196 (39.1)	24 (31.2)	140 (38.9)	32 (50)	
Male, *n* (%)	305 (60.9)	53 (68.8)	220 (61.1)	32 (50)	
CKD Stage group					0.07
CKD 1 + 2, *n* (%)	163 (32.5)	27 (35.1)	125 (34.7)	11 (17.2)	
CKD 3, *n* (%)	244 (48.7)	37 (48.1)	171 (47.5)	36 (56.3)	
CKD 4 + 5, *n* (%)	94 (18.8)	13 (16.9)	64 (17.8)	17 (26.6)	
eGFR ml/min/1.73 m^2^	51.57 (22)	52.48 (21.7)	52.68 (22.3)	44.21 (19.5)	0.02
CKD Etiology					0.007
Glomerular, *n* (%)	125 (25)	29 (37.7)	77 (21.4)	19 (29.7)	
Nonglomerular, *n* (%)	376 (75.1)	48 (62.3)	283 (78.6)	45 (70.3)	
CKD duration at ABPM in years, mean (SD)	10.3 (4.7)	9 (4.8)	10.8 (4.6)	9.4 (4.9)	0.002
BMI kg/m^2^, mean (SD)	20.5 (5.3)	22.5 (7.5)	20 (4.6)	21.1 (5.1)	0.0007
BMI Z-score, mean (SD)	0.35 (1.1)	0.72 (1.1)	0.23 (1.1)	0.53 (1.04)	0.0006
Obesity, *n* (%)	74 (14.8)	20 (26.3)	43 (12)	11 (17)	0.006
Weight Z-score, mean (SD)	0.046 (1.3)	0.48 (1.3)	− 0.50 (1.2)	0.076 (1.2)	0.003
Height Z-Score, (mean (SD)	− 0.474 (1.2)	− 0.33 (1.1)	− 0.45 (1.2)	− 0.75 (1)	0.08
Antihypertensive use					0.9
Yes, *n* (%)	329 (65.7)	50 (64.9)	235 (65.3)	44 (68.8)	
No, *n* (%)	172 (34.3)	27 (25.1)	125 (34.7)	20 (31.2)	
ACEI or ARB use					
Yes, *n* (%)	281(56)	37 (48)	202 (56.1)	42 (65.6)	0.1
No, *n* (%)	220 (44)	40 (52)	158 (43.9)	22 (34.4)	
Diuretic use					
Yes, *n* (%)	34 (6.8)	7 (9.1)	23 (6.4)	4 (6.3)	0.7
No, *n* (%)	467 (93.2)	70 (90.9)	337 (93.6)	60 (93.8)	

*ABPM*, ambulatory blood pressure monitor; *SD*, standard deviation; *CKD*, chronic kidney disease; *eGFR*, estimated glomerular filtration rate; *BMI*, body mass index; *ACEI*, angiotensin converting enzyme inhibitor; *ARB*, angiotensin receptor blocker. Obesity defined as BMI ≥ 95th percentile. *For comparison across racial-ethnic groups using anaysis of variance (ANOVA), chi-square, and Fischer exact as indicated

### Characteristics of first ambulatory blood pressure measurement

When comparing the first ABPM report for each participant (Supplemental Table 1), mean wake systolic blood pressure (SBP) differed among the groups with Black participants having the highest mean (SD) value (120.3 (11.9) mmHg in Black, 116.9 (11) mmHg in White, 116.2 (11.6) mmHg in Hispanic (*p* = 0.04)). During waking hours, 15% of the cohort experienced systolic hypertension (abnormal wake systolic mean), as defined by reference values, with Black participants demonstrating the highest frequency of wake systolic hypertension among the racial-ethnic groups (26% in Black, 13.3% in White, 14.1% in Hispanic (*p* = 0.02)). Twenty-two percent of the cohort experienced diastolic hypertension during sleep hours with Black participants again demonstrating the highest frequency of sleep diastolic hypertension among the racial-ethnic groups (31.2% in Black, 20.8% in White, 14.1% in Hispanic (*p* = 0.04)). The frequency of abnormal systolic and diastolic dipping (non-dipping) was highest in Black participants (53.3% in Black, 37.2% in White, 31.3% in Hispanic (*p* = 0.001) for systolic non-dipping and 28.6% in Black, 11.4% in White, and 10.9% in Hispanic (*p* = 0.003) for diastolic non-dipping).

### Comparisons of blood pressure measurements while awake, repeated measures regression

#### Mean blood pressure, reference-defined hypertension, and blood pressure load

**Fig. 1:**
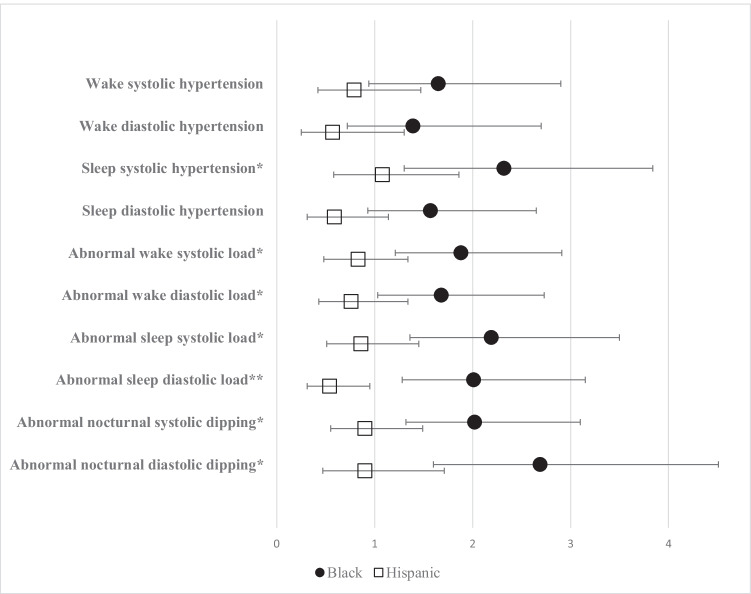
Racial-ethnic differences in hypertension outcomes, odds ratios. Reference: White. *Significant for Black cohort. **Significant for Hispanic cohort and Black cohort

In fully adjusted models, there were no differences in mean wake systolic and diastolic blood pressures in Black or Hispanic subjects as compared to White subjects (Table 2). When defining wake systolic and diastolic hypertension using reference values, there were no differences between the groups in the final models (Fig. 1, Supplemental Table 2). Black participants demonstrated higher odds of abnormal systolic load while awake (*OR* 1.88 (*CI* 1.21, 2.91, *p* = 0.005)) and abnormal diastolic load while awake (*OR* 1.69 (*CI* 1.03, 2.73 *p* = 0.04)) when compared to White participants. There were no differences in awake blood pressure load between Hispanic and White participants (Fig. 1, Supplemental Table 2).

**Table 2:** The association of racial-ethnic group with ambulatory blood pressure monitor outcomes, mean differences

Ref = White	Black	Hispanic
Wake systolic adjusted mean, mmHg (95% *CI*)		
Model 1	2.66 (0.26, 5.05), *p* = 0.03*	− 0.68 (− 3.21, 1.84), *p* = 0.6
Model 2	1.87 (− 0.57, 4.21), *p* = 0.1	− 0.89 (− 3.42, 1.64), *p* = 0.5
Model 3	1.80 (− 0.65, 4.26), *p* = 0.2	− 0.91 (− 3.46, 1.63), *p* = 0.5
Wake diastolic adjusted mean, mmHg (95% *CI*)		
Model 1	1.17 (− 0.72, 3.06), *p* = 0.2	− 1.62 (− 3.61, 0.37), *p* = 0.1
Model 2	1.35 (− 0.58, 3.29), *p* = 0.2	− 1.76 (− 3.76, 0.25), *p* = 0.09
Model 3	1.22 (− 0.72, 3.16), *p* = 0.2	− 1.79 (− 3.80, 0.22), *p* = 0.08
Sleep systolic adjusted mean, mmHg (95% *CI*)		
Model 1	4.89 (2.46, 7.33), *p* < 0.001*	− 0.99 (− 3.55, 1.56), *p* = 0.5
Model 2	4.21 (1.74, 6.69), *p* = 0.001*	− 1.02 (− 3.57, 1.53), *p* = 0.4
Model 3	4.15 (1.67, 6.64), *p* = 0.001*	− 1.04 (− 3.61, 1.53), *p* = 0.4
Sleep diastolic adjusted mean, mmHg (95% *CI*)		
Model 1	2.90 (1.09, 4.70), *p* = 0.002*	− 1.49 (− 3.39, 0.40), *p* = 0.1
Model 2	2.78 (0.95, 4.62), *p* = 0.003*	− 1.85 (− 3.74, 0.04), *p* = 0.05
Model 3	2.69 (0.85, 4.54), *p* = 0.004*	− 1.87 (− 3.78, 0.03), *p* = 0.05

*CI*, confidence interval. Model 1: age, gender. Model 2: age, gender, BMI, height z-score, CKD stage, CKD etiology. Model 3: age, gender, BMI, height z-score, CKD stage, CKD etiology, antihypertensive use. *Statistically significant outcomes

### Comparisons of blood pressure measurements while asleep, repeated measures regression

#### Mean blood pressure and reference-defined hypertension

In fully adjusted models of mean systolic blood pressure during sleep, Black race was associated with 4.2 mmHg higher sleep systolic blood pressure and 2.7 mmHg higher sleep diastolic blood pressure when compared to White participants (Table 2). In fully adjusted regression models of sleep hypertension, as defined by reference values, Black participants had higher odds of sleep systolic hypertension when compared to White participants (2.32 (1.3–3.84, *p* = 0.001)) (Supplemental Table 2). There was no difference in sleep blood pressure or reference-defined hypertension between Hispanic and White participants (Table 2, Supplemental Table 2).

#### Blood pressure load and nocturnal dipping

Black participants demonstrated higher odds of both abnormal sleep systolic load (*CI* 2.19 (1.36–3.50, *p* = 0.001)) and abnormal sleep diastolic load (*CI* 2.01 (1.28–3.15, *p* = 0.002)) as compared to White participants. Hispanic participants demonstrated *lower* odds of abnormal sleep diastolic load when compared to White participants with an *OR* (*CI*) of 0.54 (0.31–0.95, *p* = 0.03) (Fig. 1, Supplemental Table 2). Black participants, when compared to White participants, had higher odds of both abnormal systolic and diastolic dipping (non-dipping). In fully adjusted models, the odds ratio (*CI*) of systolic non-dipping in Black participants as compared to White participants was 2.02 (1.31–3.10, *p* = 0.001) and the odds ratio (*CI*) of diastolic non-dipping was 2.69 (1.60–4.51, *p* < 0.001). There was no difference in the odds of systolic or diastolic non-dipping in the Hispanic cohort as compared to the White cohort (Fig. 1, Supplemental Table 2).

### Sensitivity analyses

In addition to replicating the findings of the repeated measures analysis, a fully adjusted, simple linear regression of single ABPM studies demonstrated additional findings of higher odds of wake diastolic hypertension (2.23 (1.03–4.83), *p* = 0.04) and sleep diastolic hypertension (2.01(1.11, 3.67), *p* = 0.02) in Black participants as compared to White participants. Additionally, when Hispanic participants were used as the reference group in a repeated measures analysis, Black participants similarly demonstrated higher odds of sleep systolic and diastolic hypertension, abnormal wake and sleep systolic load, and abnormal systolic and diastolic dipping when compared to Hispanic participants (data not shown). Sensitivity analyses evaluating the impact of BMI z score instead of absolute BMI attenuated the association of higher wake diastolic load in Black participants, which was no longer significantly higher in Black as compared to White participants (*p* = 0.07). All other outcomes were unchanged. Similarly, findings were mostly unchanged when using obesity category (yes/no) instead of absolute BMI. Differences included wake diastolic load which was no longer significantly higher in Black participants (*p* = 0.05). Otherwise, findings noted in the primary analysis using absolute BMI were replicated in this analysis using obesity as a categorical variable. Finally, the use of eGFR instead of CKD stage in the original models did not change the results.

## Discussion

In this study, we demonstrate that Black pediatric patients with chronic kidney disease have higher mean nocturnal systolic and diastolic blood pressures and higher rates of nocturnal systolic hypertension when compared to White pediatric participants. Even though the absolute difference in mean blood pressure may not appear clinically significant, the diagnosis based on normative values does suggest differences in rates of hypertension between the Black and White participants. We also demonstrate higher rates of abnormal nocturnal load as well as abnormal dipping (non-dipping) within the Black cohort. Aside from *lower* odds of abnormal nocturnal diastolic load, we did not note major differences between White and Hispanic pediatric patients with CKD.

Our findings are consistent with several adult studies which describe higher rates of abnormal ABPM, specifically nocturnal hypertension and non-dipping in Black, non-CKD, adult patients [7–9, 18, 19]. Additionally, adult studies of patients with CKD have shown that Black patients have poorer blood pressure control as compared to White patients with CKD [20]. A 15-year longitudinal study of ABPM in healthy children and adolescents (ages 7 to 30 years) revealed higher daytime and nighttime blood pressure and lower nocturnal decline in blood pressure in Black adolescents as compared to White adolescents [9]. Similar to our findings, these differences were more pronounced at night. This predilection for racial differences in nocturnal blood pressure requires consideration of the impact of sleep quality on hypertension. This is especially important given prior studies demonstrating both higher rates of nocturnal hypertension and poor sleep quality and a relationship between the two in Black, non-CKD patients [21]. In addition, nocturnal hypertension may represent a compensatory response to diminished daytime natriuresis [22]. In fact, Harshfield et al. demonstrated racial differences in sodium excretion during periods of stress with Black adolescents, when compared to White, having higher average systolic blood pressure and lower sodium excretion across multiple stressful tasks [23]. When investigating salt excretion within 118 Black normotensive adolescents, Harshfield et al. demonstrated that, following a prolonged stressor, those who were able to excrete sodium had resolution of stress-induced elevations in blood pressure. Contrarily, those who retained sodium maintained a diastolic blood pressure that was significantly higher than their pre-stress blood pressure. Thus, the finding of nocturnal hypertension may represent a physiologic response to diminished salt natriuresis as previously described in Black adolescents [24].

The association of nocturnal hypertension with intermediate cardiovascular outcome underscores the clinical importance of racial differences in nocturnal hypertension. When compared to normotensive children, Seeman et al. demonstrated that children with isolated nocturnal hypertension have higher age-adjusted LVMI with nighttime systolic hypertension being the most important predictor of LVMI [25]. Furthermore, when compared to children who experienced both daytime and nocturnal hypertension, children with isolated nocturnal hypertension had a similar prevalence of left ventricular hypertrophy (LVH). These findings highlight the clinical implications of nocturnal hypertension even when in isolation. In fact, in pediatric patients with CKD III–V from the Cardiovascular Comorbidity in Children with CKD Study (4C), nocturnal hypertension was an independent predictor of left ventricular hypertrophy (LVH), carotid artery intima-media thickness (cIMT), and pulse wave velocity (PWV) [26]. Furthermore, the long-term implications of nocturnal hypertension were described by Yano et al. who showed that nighttime systolic blood pressure predicted 24% greater risk of all-cause mortality for every one standard deviation increase [27]. Thus, racial differences in nocturnal hypertension carry great risk of driving racial differences in intermediate and long-term cardiovascular outcomes. Consistent with this is the finding by Sgambat et al. demonstrating greater left ventricular mass index (LVMI) in Black children within the CKiD study [28].

Sgambat et al. also investigated racial differences in ABPM within the CKiD population and similarly showed an association between Black race and higher odds of ambulatory hypertension defined as a mean blood pressure at or above the 95th percentile or blood pressure load above 25% [28]. This association was seen only in the non-glomerular CKD group and was absent in the glomerular group. Contrary to Sgambat et al., our findings persisted even after adjustment for CKD etiology. Thus, the impact of CKD etiology deserves further investigation especially given the higher prevalence of glomerular disease within Black participants within our study. Nuances within the category of glomerular disease such as a differential prevalence of systemic lupus erythematosis may contribute to residual confounding. Sgambat et al. also noted a difference in the use of angiotensin converting enzyme inhibitors (ACEI) and angiotensin receptor blockers (ARB) between Black and White children in the CKiD population. They reported a significantly lower frequency (33%) of use in Black children versus White children (45%). Although not reaching significance in our study, we similarly found a lower frequency of ACE or ARB use in Black children. This systematic difference in the use of medications proven to be effective for blood pressure control and favored for their renoprotection is an important source of the differences noted in blood pressure within our study. Not only may it explain differences in blood pressure control but it also serves as a potential representation of prescribing bias by race [29].

Although this study is not able to assess differences in socio-economic status and its impact on access to medical care, or access to medications and environmental stressors impacting disease, it is critical to consider broader social determinants of health, especially given recent data demonstrating the role of socioeconomic status in disparate cardiovascular outcomes within pediatric CKD [28]. In fact, Sgambat et al. demonstrated an attenuation of racial differences in ambulatory hypertension and left ventricular mass index upon adjustment for numerous socioeconomic factors including household income, birth history, maternal age at birth, and food insecurity. They additionally highlighted the disproportionate experience of socioeconomic disadvantages among Black children with CKD as compared to White children with CKD. Given this and the inability to fully quantify the enormity of system racism, it is highly likely that much of the residual difference within this study is mediated by unmeasured socioeconomic disparities.

Despite the higher rates of hypertension in Hispanic children within the general population [30], we did not show a difference in rates of hypertension between White and Hispanic pediatric CKD patients. This lack of difference may reflect our small sample size. Additionally, this may reflect a difference in the etiology of hypertension between the CKD population and the general population, in which essential hypertension, often associated with obesity, is the major etiology. Given a known higher prevalence of obesity in Hispanic children within the general population [31], and the lack of a difference in the frequency of obesity between White and Hispanic children within our CKD population, the inability to detect differences in ABPM parameters may reflect similar rates of obesity. Contrary to Hispanic children, Black children had a significantly higher prevalence of obesity as compared to White children. Although adjustment for obesity did not significantly change the primary findings, knowing the important connection between obesity and hypertension, we cannot rule out residual confounding related to differences in the prevalence of obesity.

Several issues relating to the interpretation of pediatric ABPM warrant discussion. When investigating rates of hypertension, it is important to consider the reference data of normative values that are used to define hypertension. In this study, the ABPM results are compared to the normative values published by Soergel et al. [16]. This database is composed of 1114 healthy, Caucasian students in Germany and has not been validated for use in non-White ethnic groups. Since no other studies of normal ABPM values are available, we may systematically overestimate or underestimate the rates of daytime and nighttime hypertension in Black and Hispanic pediatric patients. The need for a larger, more diverse database of normal ABPM values has been previously highlighted by Flynn et al. [32]. An additional issue is the use of blood pressure load to define hypertension—a practice unique to pediatric hypertension guidelines. Underscoring the questionability of this practice is a repeated lack of association between blood pressure load and left ventricular hypertrophy in both CKD and healthy adolescent populations [33, 34]. Given that blood pressure load does not provide better prediction of outcomes beyond that of mean blood pressure, the findings of racial-ethnic differences in blood pressure load may not carry much prognostic significance.

We do appreciate that this study has limitations. One major limitation is the sample size, overall, and, specifically, the representation of minority groups. With a larger sample size, it is possible that a difference in wake hypertension rates would be present. It is also possible that a larger sample size would reflect a difference in hypertension rates between Hispanic and White patients. Given the small sample size, we did not exclude patients taking medications, like steroids, which might elevate blood pressure. This is an additional potential source of confounding. Additionally, our study uses 95th percentiles as defined by Soergel et al. whereas there is a more recent shift toward the use of percentiles defined by Wuhl et al. [17]. These percentiles were unavailable in the public database and therefore were not used in this study. Use of the public CKiD database also poses a limitation given the data does not represent the most up-to-date ABPM data available in the CKiD study. Although ABPM use was part of the CKiD protocol, contemporary advances in ABPM recommendations and ABPM availability for pediatric patients with CKD may have contributed to increased ABPM uptake in recent years not reflected by our study. Finally, in our study, Black children had a lower frequency of ABPM use overall and lower frequency of having more than one ABPM. This may reflect social barriers to routine follow-up or biases limiting the use of routine resources within this population. Regardless of the etiology, there may be selection bias such that those with severe hypertension were more likely to be offered and successfully complete an ABPM than children with normal blood pressure. Such bias could contribute to the greater blood pressure seen among the Black participants.

Despite these limitations, this is one of few studies demonstrating racial-ethnic differences in ABPM results in a pediatric population with CKD. This study analyzed a wide variety of ABPM measurements and had a sample of patients from multiple pediatric nephrology centers in North America. Given our findings, it is important to consider potentially modifiable contributors to these differences and the clinical implications on long-term cardiovascular health within pediatric CKD.

## Supplementary Information

Below is the link to the electronic supplementary material.

Supplementary file1 (PPTX 58 KB)

Supplementary file2 (DOCX 54 KB)

## Data Availability

All data used in the performance of this research is publicly available at the NIH Central Repository-The Chronic Kidney Disease in Children Study.

## References

[1] Lewington S, Clarke R, Qizilbash N, Peto R, Collins R, Collaboration PS (2002). Age-specific relevance of usual blood pressure to vascular mortality: a meta-analysis of individual data for one million adults in 61 prospective studies. Lancet.

[2] Song P, Zhang Y, Yu J, Zha M, Zhu Y, Rahimi K, Rudan I (2019). Global prevalence of hypertension in children: a systematic review and meta-analysis. JAMA Pediatr.

[3] Rao G (2016). Diagnosis, epidemiology, and management of hypertension in children. Pediatrics.

[4] Ku E, Lee B, Wei J, Weir M (2019). Hypertension in CKD: core curriculum 2019. Am J Kidney Dis.

[5] Samuels J, Ng D, Flynn J, Mitsnefes M, Poffenbarger T, Warady B, Furth S (2012). Ambulatory blood pressure patterns in children with chronic kidney disease. Hypertension.

[6] Mitsnefes M, Flynn J, Cohn S, Samuels J, Blydt-Hansen T, Saland J, Kimball T, Furth S, Warady B (2010). Masked hypertension associates with left ventricular hypertrophy in children with CKD. J Am Soc Nephrol.

[7] Muntner P, Lewis C, Diaz K, Carson A, Kim Y, Calhoun D, Yano Y, Viera A, Shimbo D (2015). Racial differences in abnormal ambulatory blood pressure monitoring measures: results from the Coronary Artery Risk Development in Young Adults (CARDIA) study. Am J Hypertens.

[8] Booth J, Anstey D, Bello N, Jaeger B, Pugliese D, Thomas S, Deng L, Shikany J, Lloyd-Jones D, Schwartz J, Lewis C, Shimbo D, Muntner P (2019). Race and sex differences in asleep blood pressure: the Coronary Artery Risk Development in Young Adults (CARDIA) study. J Clin Hypertens (Greenwich).

[9] Wang X, Poole J, Treiber F, Harshfield G, Hanevold CD, Snieder H (2006). Ethnic and gender differences in ambulatory blood pressure trajectories: results from a 15-year longitudinal study in youth and young adults. Circulation.

[10] Hertz R, Unger A, Cornell J, Saunders E (2005). Racial disparities in hypertension prevalence, awareness, and management. Arch Intern Med.

[11] Kit B, Kuklina E, Carroll M, Ostchega Y, Freedman D, Ogden C (2015). Prevalence of and trends in dyslipidemia and blood pressure among US children and adolescents, 1999–2012. JAMA Pediatr.

[12] Hardy S, Sakhuja S, Jaeger B, Urbina E, Suglia S, Feig D, Muntner P (2021). Trends in blood pressure and hypertension among US children and adolescents, 1999–2018. JAMA Netw Open.

[13] National Institute of Diabetes and Digestive and Kidney Diseases. NIDDK Central Repository. The Chronic Kidney Disease in Children Cohort Study (CKiD). https://repository.niddk.nih.gov/studies/ckid/. Accessed 20 Nov 2019

[14] Furth S, Cole S, Moxey-Mims M, Kaskel F, Mak R, Schwartz G, Wong C, Munoz A, Warady B (2006). Design and methods of the Chronic Kidney Disease in Children (CKiD) prospective cohort study. Clin J Am Soc Nephrol.

[15] Schwartz G, Haycock G, Edelmann C, Spitzer A (1976). A simple estimate of glomerular filtration rate in children derived from body length and plasma creatinine. Pediatrics.

[16] Soergel M, Kirschstein M, Busch C, Danne T, Gellermann J, Holl R, Krull F, Reichert H, Reusz G, Rascher W (1997). Oscillometric twenty-four-hour ambulatory blood pressure values in healthy children and adolescents: a multicenter trial including 1141 subjects. J Pediatr.

[17] Wuhl E, Witte K, Soergel M, Mehls O, Schaefer F, German Working Group in Hypertension (2002). Distribution of 24-h ambulatory blood pressure in children: normalized reference values and role of body dimensions. J Hypertension.

[18] Profant J, Dimsdale J (1999). Race and diurnal blood pressure patterns. A review and meta-analysis. Hypertension.

[19] Husain A, Lin F, Tuttle L, Olsson E, Viera A (2017). The reproducibility of racial differences in ambulatory blood pressure phenotypes and measurements. Am J Hypertens.

[20] Duru O, Li S, Jurkovitz C, Bakris G, Brown W, Chen S, Collins A, Klag M, McCullough P, McGill J, Narva A, Pergola P, Singh A, Norris K (2008). Race and sex differences in hypertension control in CKD: results from the Kidney Early Evaluation Program (KEEP). Am J Kidney Dis.

[21] Sherwood A, Routledge F, Wohlgemuth W, Hinderliter A, Kuhn C, Blumenthal J (2011). Blood pressure dipping: ethnicity, sleep quality, and sympathetic nervous system activity. Am J Hypertens.

[22] Kimura G, Dohi Y, Fukuda M (2010). Salt sensitivity and circadian rhythm of blood pressure: the keys to connect CKD with cardiovascular events. Hypertens Res.

[23] Harshfield G, Trieber F, Davis H, Kapuku G (2002). Impaired stress-induced pressure natriuresis is related to left ventricle structure in Blacks. Hypertension.

[24] Harshfield G, Wilson M, Hanevold C, Kapuku G, Mackey L, Gillis D, Treiber F (2002). Impaired stress-induced pressure natriuresis increases cardiovascular load in African American youths. Am J Hypertens.

[25] Seeman T, Hradsky O, Gilik J (2021). Isolated nocturnal hypertension is associated with increased left ventricular mass index in children. Pediatr Nephrol.

[26] Duzova A, Karabay Bayazit A, Canpolat N, Niemirska A, Kaplan Bulut I, Azukaitis K, Karagoz T, Oguz B, Erdem S, Anarat A, Ranchin B, Shroff R, Djukic M, Harambat J, Yilmaz A, Yildiz N, Ozcakar B, Buscher A, Lugani F, Wygoda S, Tschumi S, Zaloszyc A, Jankauskiene A, Laube G, Galiano M, Kirchner M, Querfeld U, Melk A, Schaefer F, Wuhl E (2019). Isolated nocturnal and isolated daytime hypertension associate with altered cardiovascular morphology and function in children with chronic kidney disease: findings from the Cardiovascular Comorbidity in Children with Chronic Kidney Disease study. J Hypertens.

[27] Yano Y, Tanner R, Sakhuja S, Jaeger B, Booth J, Abdalla M, Pugliese D, Seals S, Ogedegbe G, Jones D, Muntner P, Shimbo D (2019). Association of daytime and nighttime blood pressure with cardiovascular disease events among African American individuals. JAMA Cardiol.

[28] Sgambat K, Roem J, Brady T, Flynn J, Mitsnefes M, Samuels J, Warady B, Furth S, Moudgil A (2021). Social determinants of cardiovascular health in African American children with CKD: an analysis of the chronic Kidney Disease in Children (CKiD) study. Am J Kidney Dis.

[29] Holt HK, Gildengorin G, Karliner L, Fontil V, Pramanik R, Potter MB (2022). Differences in hypertension medication prescribing for Black Americans and their association with hypertension outcomes. J Am Board Fam Med.

[30] Din-Dzietham R, Liu Y, Bielo MV, Shamsa F (2007). High blood pressure trends in children and adolescents in national surveys, 1963 to 2002. Circulation.

[31] Hales C, Carroll M, Fryar C, Ogden C (2017) Prevalence of obesity among adults and youth: United States, 2015–2016. NCHS Data Brief 288:1–8 29155689

[32] Flynn J (2011) Ambulatory blood pressure monitoring in children: imperfect yet essential. Pediatr Nephrol 26:2089–2094 10.1007/s00467-011-1984-9 21866381

[33] Lee J, McCulloch CE, Flynn JT, Samuels J, Warady BA, Furth SL, Seth D, Grimes BA, Mitsnefes MM, Ku E (2020). Prognostic value of ambulatory blood pressure load in pediatric CKD. Clin J Am Soc Nephrol.

[34] Hamdani G, Mitsnefes MM, Flynn JT, Becker RC, Daniels S, Falkner B, Ferguson M, Hooper S, Hanevold C, Ingelfinger J, Lande M, Martin L, Meyers K, Rosner B, Samuels J, Urbina E (2021). Pediatric and adult ambulatory blood pressure thresholds and blood pressure load as predictors of left ventricular hypertrophy in adolescents. Hypertension.

