# Supplementary material for: Racial-ethnic diversity in ambulatory blood pressure monitoring in children with chronic kidney disease
Source: Pediatr Nephrol. 2022 Jul 8;38(3):819–27. doi: 10.1007/s00467-022-05659-2 (PMC9842582; doi:10.1007/s00467-022-05659-2)
Supplement: Supplementary file 1 — Supplementary file1 (PPTX 58 KB) [file 467_2022_5659_MOESM1_ESM.pptx]

## Slide 1
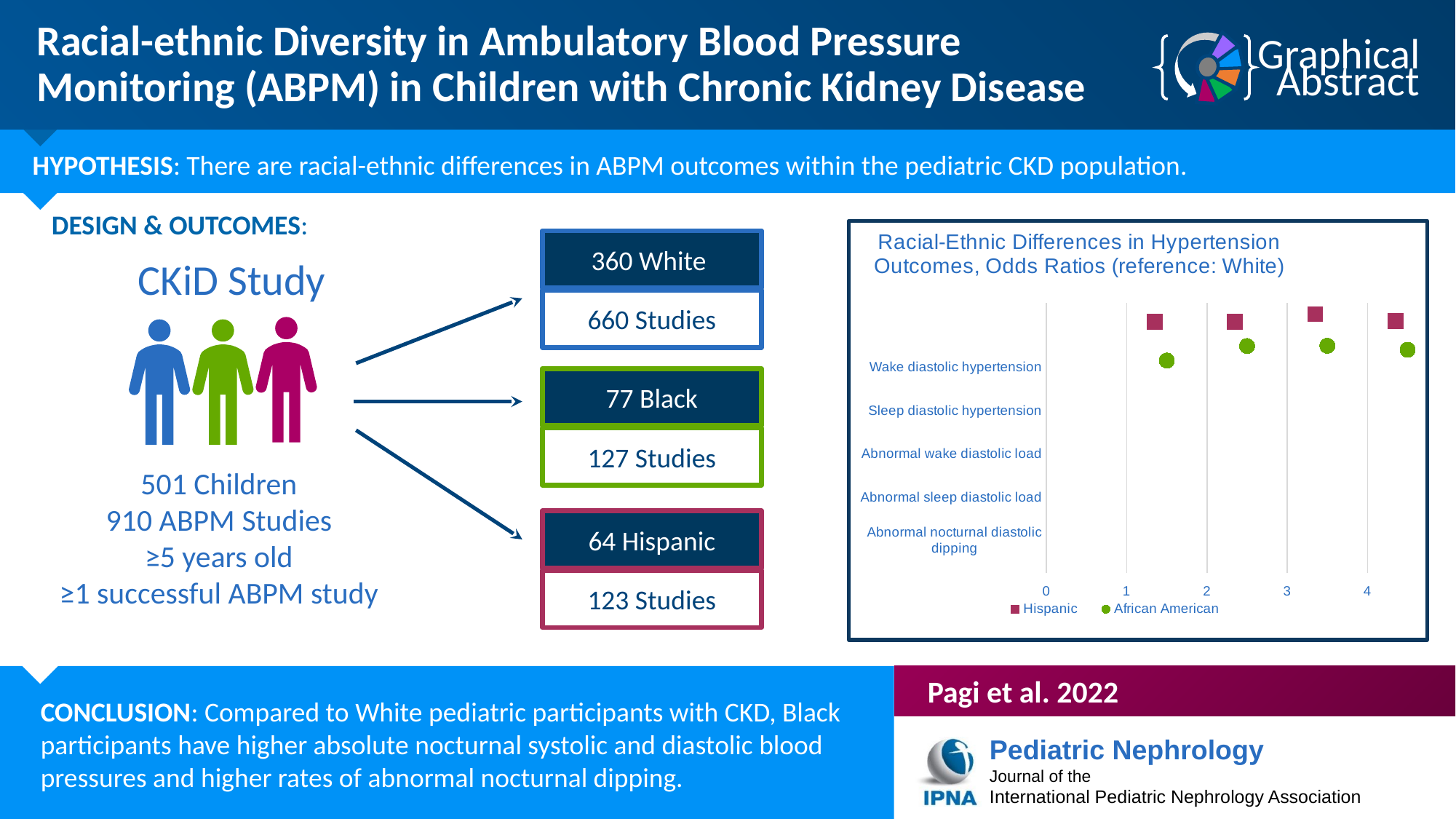

Racial-ethnic Diversity in Ambulatory Blood Pressure
Monitoring (ABPM) in Children with Chronic Kidney Disease
HYPOTHESIS: There are racial-ethnic differences in ABPM outcomes within the pediatric CKD population.
DESIGN & OUTCOMES:
### Chart: Racial-Ethnic Differences in Hypertension Outcomes, Odds Ratios (reference: White)
| Category | | African American | Hispanic |
|---|---|---|---|
| | None | None | None |
| Wake systolic hypertension | 10.5 | 10.5 | 10.35 |
| Wake diastolic hypertension | 9.5 | 9.5 | 9.35 |
| Sleep systolic hypertension | 8.5 | 8.5 | 8.35 |
| Sleep diastolic hypertension | 7.5 | 7.5 | 7.35 |
| Abnormal wake systolic load | 6.5 | 6.5 | 6.35 |
| Abnormal wake diastolic load | 5.5 | 5.5 | 5.35 |
| Abnormal sleep systolic load | 4.5 | 4.5 | 4.35 |
| Abnormal sleep diastolic load | 3.5 | 3.5 | 3.35 |
| Abnormal nocturnal systolic dipping | 2.5 | 2.5 | 2.35 |
| Abnormal nocturnal diastolic dipping | 1.5 | 1.5 | 1.35 |360 White
CKiD Study
660 Studies
77 Black
127 Studies
501 Children
910 ABPM Studies
≥5 years old
≥1 successful ABPM study
64 Hispanic
123 Studies
Pagi et al. 2022
CONCLUSION: Compared to White pediatric participants with CKD, Black participants have higher absolute nocturnal systolic and diastolic blood pressures and higher rates of abnormal nocturnal dipping.
