# Supplementary material for: Racial-ethnic diversity in ambulatory blood pressure monitoring in children with chronic kidney disease
Source: Pediatr Nephrol. 2022 Jul 8;38(3):819–27. doi: 10.1007/s00467-022-05659-2 (PMC9842582; doi:10.1007/s00467-022-05659-2)
Supplement: Supplementary file 2 — Supplementary file2 (DOCX 54 KB) [file 467_2022_5659_MOESM2_ESM.docx]

**Supplemental Figure 1** Construction of the study population

891 CKID Subjects

195 subjects did

not have ABPM

696 subjects who had ABPM

74 subjects had

unsuccessful ABPM

622 subjects with successful ABPM

(1,140 observations)

113 subjects of ethnicity other than study groups

509 Black, White,

and Hispanic subjects (933 observations)

7 subjects < 5 years old

501 subjects $\geq5$ years old (910 observations)

64 Hispanic subjects

(123 observations)

77 Black subjects

(127 observations)

360 White subjects

(660 observations)

| **Supplemental Table 1** Unadjusted blood pressure outcomes at the first ABPM visit by race and ethnicity | | | | | |
| --- | --- | --- | --- | --- | --- |
|  | **Total** | **Black** | **White** | **Hispanic** | **P value*** |
| Wake SBP mmHg, mean (SD) | 117.3 (11.3) | 120.3 (11.9) | 116.9 (11) | 116.2 (11.6) | 0.04 |
| Wake DBP mmHg, mean (SD) | 72.2 (8.4) | 73.4 (8.6) | 72.1 (8.1) | 71.1 (9.7) | 0.3 |
| Sleep SBP mmHg, mean (SD) | 104.3 (11.6) | 108.9 (11.1) | 103.6 (11.6) | 102.5 (10.6) | 0.0006 |
| Sleep DBP mmHg, mean (SD) | 59.8 (8.5) | 62.5 (8.5) | 59.4 (8.4) | 58.3 (8.8) | 0.006 |
| Wake systolic mean |  |  |  |  | 0.02 |
| Normal, n (%) | 424 (84.6) | 57 (74) | 312 (86.7) | 55 (85.9) |  |
| Abnormal, n (%) | 77 (15.4) | 20 (26) | 48 (13.3) | 9 (14.1) |  |
| Wake diastolic mean |  |  |  |  | 0.4 |
| Normal, n (%) | 446 (89) | 65 (84.4) | 324 (90) | 57 (89.1) |  |
| Abnormal, n (%) | 55 (11) | 12 (15.6) | 36 (10) | 7 (10.9) |  |
| Sleep systolic mean |  |  |  |  | 0.05 |
| Normal, n (%) | 407 (81.2) | 55 (71.4) | 300 (83.3) | 52 (81.3) |  |
| Abnormal, n (%) | 94 (18.8) | 22 (28.6) | 60 (16.7) | 12 (18.7) |  |
| Sleep diastolic mean |  |  |  |  | 0.04 |
| Normal, n (%) | 393 (78.4) | 53 (68.8) | 285 (79.2) | 55 (85.9) |  |
| Abnormal, n (%) | 108 (21.6) | 24 (31.2) | 75 (20.8) | 9 (14.1) |  |
| Wake systolic load |  |  |  |  | 0.03 |
| Normal, n (%) | 332 (66.3) | 41 (53.3) | 248 (68.9) | 43 (67.2) |  |
| Abnormal, n (%) | 169 (33.7) | 36 (46.8) | 112 (31.1) | 21 (32.8) |  |
| Wake diastolic load |  |  |  |  | 0.1 |
| Normal, n (%) | 349 (69.7) | 46 (59.7) | 257 (71.4) | 46 (71.9) |  |
| Abnormal, n (%) | 152 (30.3) | 31 (40.3) | 103 (28.6) | 18 (28.1) |  |
| Sleep systolic load |  |  |  |  | 0.004 |
| Normal, n (%) | 318 (63.5) | 36 (46.8) | 238 (66.1) | 44 (68.8) |  |
| Abnormal, n (%) | 183 (36.5) | 41 (53.3) | 122 (33.9) | 20 (31.3) |  |
| Sleep diastolic load |  |  |  |  | 0.0001 |
| Normal, n (%) | 296 (59.1) | 30 (39) | 220 (61.1) | 46 (71.9) |  |
| Abnormal, n (%) | 205 (40.9) | 47 (61) | 140(38.9) | 18 (28.1) |  |
| Systolic Dipping |  |  |  |  | 0.001 |
| Normal, n (%) | 306 (61.1) | 36 (46.8) | 226 (62.8) | 44 (68.8) |  |
| Abnormal, n (%) | 195 (38.9) | 41 (53.3) | 134 (37.2) | 20 (31.3) |  |
| Diastolic Dipping |  |  |  |  | 0.003 |
| Normal, n (%) | 431 (86) | 55 (71.4) | 319 (88.6) | 57 (89.1) |  |
| Abnormal, n (%) | 70 (14) | 22 (28.6) | 41 (11.4) | 7 (10.9) |  |

SBP, Systolic Blood Pressure; DBP, Diastolic Blood Pressure

* For comparison across racial-ethnic groups using Analysis of Variance (ANOVA), Chi-Square and Fischer exact as indicated.

| **Supplemental Table 2** The Association of Racial-ethnic Group with Ambulatory Blood Pressure Monitor Outcomes, Odds Ratio | | |
| --- | --- | --- |
| **ref=White** | **Black vs. White** | **Hispanic vs. White** |
| Presence of wake systolic hypertension^a^, OR (95% CI) |  |  |
| Model 1 | 1.85 (1.09,3.15), p=0.02* | 0.93 (0.50,1.71), p=0.8 |
| Model 2 | 1.66 (0.96,2.91), p=0.08 | 0.79 (0.42,1.47), p=0.5 |
| Model 3 | 1.65 (0.94,2.90), p=0.08 | 0.79 (0.42,1.47), p=0.5 |
| Presence of wake diastolic hypertension, OR (95% CI) |  |  |
| Model 1 | 1.43 (0.79,2.62), p=0.2 | 0.73 (0.34,1.57), p=0.4 |
| Model 2 | 1.44 (0.75,2.75), p=0.3 | 0.57 (0.25,1.31), p=0.2 |
| Model 3 | 1.39 (0.72,2.70), p=0.3 | 0.57 (0.25,1.30), p=0.2 |
| Presence of sleep systolic hypertension, OR (95% CI) |  |  |
| Model 1 | 2.29 (1.42,3.69), p<0.001* | 1.09 (0.60,1.97), p=0.8 |
| Model 2 | 2.26 (1.37,3.76), p=0.002 | 1.06 (0.58,1.97), p=0.8 |
| Model 3 | 2.32 (1.30,3.84), p=0.001* | 1.08 (0.58,1.86), p=0.8 |
| Presence of sleep diastolic hypertension, OR (95% CI) |  |  |
| Model 1 | 1.54 (0.95,2.50), p=0.08 | 0.69 (0.38,1.25), p=0.2 |
| Model 2 | 1.58 (0.95,2.65), p=0.08 | 0.59 (0.31,1.14), p=0.1 |
| Model 3 | 1.57 (0.93,2.65), p=0.09 | 0.59 (0.31,1.14), p=0.1 |
| Presence of abnormal wake systolic load, OR(95% CI) |  |  |
| Model 1 | 1.95 (1.26,3.01), p=0.003* | 0.94 (0.57,1.57), p=0.8 |
| Model 2 | 1.90 (1.20,2.88), p=0.005* | 0.81 (0.48,1.35), p=0.4 |
| Model 3 | 1.88 (1.21,2.91), p=0.005* | 0.83 (0.48,1.34), p=0.4 |
| Presence of abnormal wake diastolic load, OR (95% CI) |  |  |
| Model 1 | 1.54 (0.97,2.44), p=0.07 | 0.82 (0.47,1.42), p=0.5 |
| Model 2 | 1.71 (1.05,2.76), p=0.03* | 0.76 (0.43, 1.35), p=0.4 |
| Model 3 | 1.68 (1.03,2.73), p=0.04* | 0.76 (0.43,1.34), p=0.3 |
| Presence of abnormal sleep systolic load, OR (95% CI) |  |  |
| Model 1 | 2.26 (1.46,3.50), p<0.001* | 0.92 (0.55,1.53), p=0.8 |
| Model 2 | 2.19 (1.37,3.50), p=0.001* | 0.86 (0.51,1.45), p=0.6 |
| Model 3 | 2.19 (1.36,3.50), p=0.001* | 0.86 (0.51,1.45), p=0.6 |
| Presence of abnormal sleep diastolic load, OR (95% CI) |  |  |
| Model 1 | 2.10 (1.38,3.18), p<0.001* | 0.63 (0.37,1.05), p=0.08 |
| Model 2 | 2.03 (1.31,3.16), p=0.002* | 0.54 (0.31,0.95), p=0.03* |
| Model 3 | 2.01 (1.28,3.15), p=0.002* | 0.54 (0.31,0.95), p=0.03* |
| Presence of abnormal nocturnal systolic dipping, OR(95% CI) |  |  |
| Model 1 | 2.09 (1.39,3,16), p<0.001* | 0.84 (0.52,1.37), p=0.5 |
| Model 2 | 2.04 (1.33,3.18), p=0.001* | 0.91 (0.55,1.50), p=0.7 |
| Model 3 | 2.02 (1.32,3.10), p=0.001* | 0.90 (0.55,1.49), p=0.7 |
| Presence of abnormal nocturnal diastolic dipping, OR (95% CI) |  |  |
| Model 1 | 3.02 (1.82,5.00), p<0.001* | 1.04 (0.57,1.88), p=0.9 |
| Model 2 | 2.64 (1.58,4.43), p<0.001* | 0.90 (0.47, 1.71), p=0.8 |
| Model 3 | 2.69 (1.60,4.51), p<0.001* | 0.90 (0.47,1.71), p=0.8 |
| OR, Odds ratio; |  |  |
| CI, confidence interval |  |  |
| Model 1: Age, gender |  |  |
| Model 2: Age, gender, BMI, height z-score, CKD stage, CKD etiology | |  |
| Model 3: Age, gender, BMI, height z-score, CKD stage, CKD etiology, antihypertensive use | | |
| ^a^All models for wake systolic hypertension include time since baseline CKiD visit. | |  |
| *statistically significant outcomes |  |  |
